# Maritime Trajectory Forecasting via CNN–SOFTS-Based Coupled Spatio-Temporal Features

**DOI:** 10.3390/s26051547

**Published:** 2026-03-01

**Authors:** Yongfeng Suo, Chunyu Yang, Gaocai Li, Qiang Mei, Lei Cui

**Affiliations:** Navigation College, Jimei University, Xiamen 361021, China

**Keywords:** spatio-temporal features, curved waterways, CNN–SOFTS–based framework, CNN model, SOFTS model, trajectory prediction

## Abstract

Spatio-temporal features are crucial for maritime trajectory forecasting, especially in scenarios involving curved waterways or abrupt changes in ship motion patterns. Although Automatic Identification System (AIS) data, which are widely used for trajectory prediction, inherently include temporal and spatial information, effectively strengthening these features and integrating them into prediction models remains challenging. To address this challenge, we propose a Convolutional Neural Network (CNN)-Series-cOre Fused Time Series forecaster (SOFTS)-based framework that explicitly couples spatial and temporal features to achieve high-fidelity maritime trajectory forecasting, especially in scenarios with complex spatial patterns. We first employ a CNN-based spatial encoder to hierarchically abstract spatial density distributions through convolution and pooling operations, thereby learning global spatial structure patterns of ship movements. This encoder emphasizes overall spatial morphology rather than precise individual trajectory points. Second, we employ the SOFTS model to incorporate angular velocity, acceleration, and angular acceleration as input features to characterize ship motion states, which can capture the temporal dependencies of ship motion states from multivariate time series. Finally, the spatial embedding features extracted by the CNN are concatenated with the temporal feature representations learned by SOFTS along the feature dimension to form a joint spatiotemporal representation. This representation is then fed into a fusion regression module composed of fully connected layers to predict future ship trajectories. Experimental results on the validation dataset show that the proposed method achieves an MSE of 0.020 and an MAE of 0.060, outperforming several advanced time series forecasting models in prediction accuracy and computational efficiency. The introduction of angular velocity, acceleration, and angular acceleration features reduces the MSE and MAE by approximately 10.22% and 9.49%, respectively, validating the effectiveness of the introduced dynamic features in improving trajectory prediction performance. These results underscore the proposed method’s potential for intelligent navigation and traffic management systems by effectively enhancing inland river navigation safety and strengthening waterborne traffic monitoring capabilities.

## 1. Introduction

As an important part of the domestic economy and logistics network, inland waterway transportation plays a key role in the transportation of bulk goods and the stabilization of regional supply chains. Against the background of increasing ship numbers and waterway density, complex inland river geomorphology and highly curved channel structures lead to pronounced variations in ship trajectory curvature. Ships operating in highly curved and constrained waterways undergo more frequent navigation state changes, which increases the uncertainty of trajectory prediction [1,2]. The evolution of inland river ship trajectories depends jointly on the spatial distribution characteristics of trajectories and the temporal dynamic features of ship motion. Therefore, spatiotemporal features play a key role in improving the accuracy of inland river ship trajectory prediction.

Ship trajectory prediction is a rapidly developing research field whose core objective is to improve the accuracy and reliability of prediction models. Early studies mainly used traditional statistics-based prediction methods to construct mathematical models to predict the future trajectories of ships by analyzing historical ship motion data. Existing studies have used methods such as Kalman filtering, Gaussian mixture models, and Bayesian networks to model and predict ship trajectories [3,4,5]. However, these early methods mainly rely on historical data and the physical characteristics of the ship. They inadequately capture the spatiotemporal features embedded in trajectories, and they exhibit limitations in modeling complex environments, nonlinear behaviors, and large-scale data. As a result, these methods fail to meet the requirements of ship trajectory prediction in increasingly complex maritime traffic environments.

The emergence of deep learning methods has provided an opportunity to delve deeper into the information within data. Deep learning prediction models widely cover Recurrent Neural Networks (RNN), Transformer models, Multilayer Perceptron (MLP) and other types of deep prediction architectures. These models can effectively mine and analyze complex patterns in ship trajectories, especially when dealing with nonlinear relationships and complex patterns.

Early research on ship trajectory prediction mainly relied on RNN [6]. However, RNN models may suffer from problems such as gradient vanishing and gradient explosion, which are usually caused by inappropriate data characteristics or poorly designed loss functions [7]. To overcome this problem, Long Short-Term Memory (LSTM) and Gated Recurrent Units (GRU) models effectively mitigate the gradient explosion and gradient vanishing problems by designing a “gate” structure. LSTM-based and GRU-based methods have been widely applied to ship trajectory prediction. These methods model AIS time-series data to achieve short-term prediction of ship motion states [8,9,10,11]. Research [12] further introduces difference variables into LSTM units to alleviate temporal dependence and improve the stability of ship trajectory prediction. However, LSTM and GRU models still face the problems of overfitting and low computational efficiency, especially when dealing with long time series data, which motivates researchers to explore more efficient model structures.

To address the performance bottlenecks encountered by LSTM and GRU models in long time-series modeling, the Transformer model has attracted increasing attention in time-series forecasting. In recent years, many studies have applied Transformer models to maritime trajectory prediction [13,14,15,16,17], in which the self-attention mechanism efficiently captures long-term dependencies. To further explore the potential of Transformer in time series forecasting, researchers have proposed various Transformer variants. The Informer model [18] is an efficient Transformer-based model that introduces the innovative ProbSparse self-attention mechanism and self-attention distilling operation. These innovations are designed to address long time series forecasting tasks. The TFformer model [19] combines frequency domain and time domain analysis by using a sequential frequency attention block to enhance important periodic information while preserving temporal dependencies. This approach improves both prediction accuracy and interpretability. The Autoformer model [20] extracts trend information gradually through a decomposition module and utilizes an Auto-Correlation mechanism to enhance information utilization, demonstrating strong performance in long time-series forecasting. However, when the number of input feature channels increases and ship navigation states undergo abrupt changes, the Transformer model often shows limited generalization ability. At the same time, the high computational complexity of self-attention restricts its application in practical engineering scenarios.

Some researchers have found that simpler models perform better than Transformer models in specific situations. This has led to increasing attention to MLP in related research. Gan et al. [21] proposed an improved MLP network in early work, which accurately predicted long-term ship speed using particle swarm optimization and orthogonal least squares. Liu et al. [22] introduced a multi-task deep learning model for predicting ship trajectories and collision risks. The model trains an MLP as a teacher network, which inputs the state information of two ships to predict their collision risk. Additionally, Ekambaram et al. [23] proposed the TSMixer model, which is based on MLP and incorporates a modular design along with online reconciliation heads. This model significantly improved the accuracy of multivariate time-series forecasting while being more computationally efficient than Transformer models. Although these methods effectively reduce the computational complexity of long sequence processing, when a ship undergoes abrupt turning maneuvers or experiences frequent speed variations during navigation, such models still exhibit limited predictive robustness in complex scenarios. Time series prediction models often focus primarily on mining information along the temporal dimension, while they insufficiently consider the spatial characteristics of overall ship navigation. Therefore, researching efficient and accurate ship trajectory prediction methods that can fully utilize the temporal and spatial features of navigation data, especially for complex navigation scenarios such as curved waterways, is of great significance for improving maritime monitoring and management capabilities.

Using the Beijing–Hangzhou Grand Canal as a case study, we propose a CNN–SOFTS [24]-based framework that explicitly couples spatial and temporal features to achieve high-fidelity ship trajectory prediction. The SOFTS model is an MLP-based time-series forecasting model designed to handle long sequences and multi-channel data. The model incorporates an innovative STAD module, which aggregates information from all channels to form a global core representation and dispatches it to individual channels, achieving indirect channel interaction. Compared with traditional attention mechanisms, this interaction mechanism offers stronger adaptability and robustness. It effectively reduces overreliance on a single channel and thereby enhances the generalization ability of multichannel time series. Meanwhile, the macro-level spatial features extracted by CNN provide overall navigation patterns of ships, further enhancing spatial feature characterization for trajectories. The main contributions of this paper are as follows:

(1) We propose a CNN–SOFTS-based framework for ship trajectory prediction. The framework employs CNN to extract the spatial density distribution features of ship trajectories and couples them with the temporal features extracted by SOFTS, thereby achieving spatio-temporal joint modeling of ship trajectories. This improves prediction accuracy in curved waterway scenarios.
(2) We introduce acceleration, angular velocity, and angular acceleration as additional temporal dynamic variables to enhance the temporal representation of ship motion, enabling the model to more accurately capture rapid motion variations and temporal dependencies in ship navigation behavior.
(3) We introduce the SOFTS model for ship trajectory prediction. The model leverages a stable cross-channel information interaction mechanism and provides a prediction approach with lower computational cost and stronger generalization ability for complex ship trajectory prediction tasks.

## 2. Study Area

**Figure 1:**
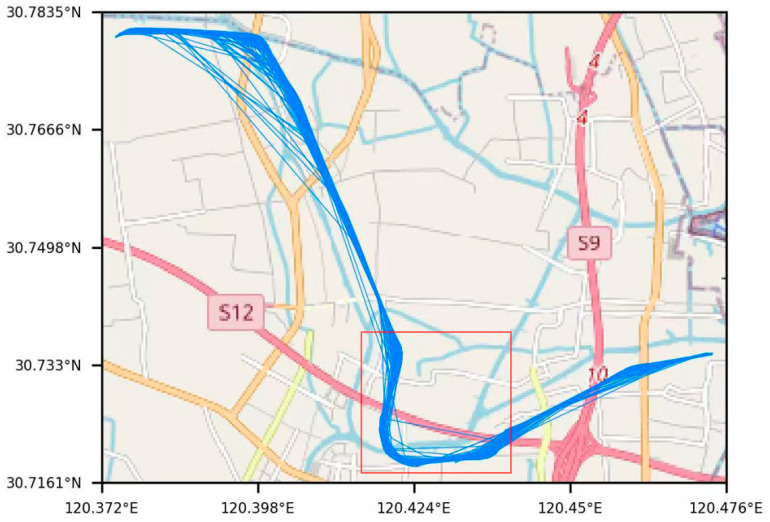
Ship trajectories in the selected reach of the Beijing–Hangzhou Canal. The figure shows cargo ship trajectories within the study area, where the red boxed region indicates a curved waterway with relatively large channel curvature. Base map data © OpenStreetMap contributors. The software version used is Python 3.11.5.

In this study, the Beijing–Hangzhou Canal, specifically the reach from Tangjiawan through Beilianli to Nanniwan, was selected as the study area, with cargo ships operating in this section serving as the research objects. After data preprocessing, we obtained AIS trajectory data for 2784 cargo ships. The dataset contains approximately 157,000 AIS trajectory points. These data provide sufficient support for model training and performance evaluation. There are several bends in this section, and the large curvature of the channel poses a potential risk to the stability and safety of ship navigation. In particular, the area marked by the red box represents a curved waterway with typical inland waterway characteristics, where the high channel curvature imposes higher requirements on ship maneuvering performance. Figure 1 presents the ship trajectories in the study area.

## 3. Methods

**Figure 2:**
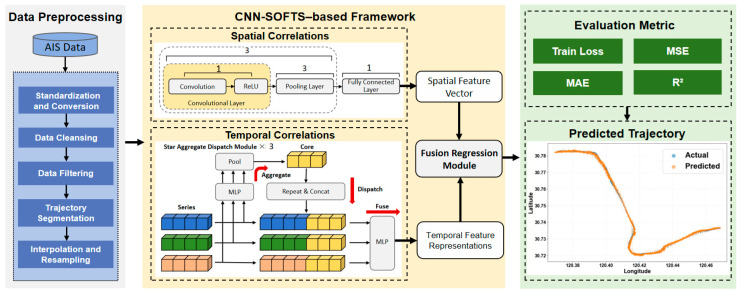
Flowchart of ship trajectory prediction, which is divided into three main parts: AIS data preprocessing, spatio-temporal feature extraction and fusion, and ship trajectory prediction.

The framework flowchart is illustrated in Figure 2. First, we preprocess the original AIS data to enhance data quality through Data Standardization and Conversion, Data Cleansing, Data Filtering, Trajectory Segmentation, and Interpolation and Resampling. Next, the acquired key ship data are fed into the CNN–SOFTS-based framework. The CNN module in the framework extracts macroscopic spatial patterns from daily trajectories and identifies overall navigation behaviors. The SOFTS module in the framework leverages its long-sequence temporal modeling capability to capture long-term dependencies in trajectory data. Subsequently, the spatial embedding features extracted by the CNN module are concatenated with the temporal feature representations learned by the SOFTS module along the feature dimension to form a joint spatiotemporal representation. This representation is then fed into a fusion regression module composed of fully connected layers to predict ship trajectories. Finally, we evaluate the proposed method using performance metrics, runtime, and predicted trajectories to comprehensively assess its effectiveness.

### 3.1. AIS Data

This study selected AIS data of the Beijing–Hangzhou Grand Canal from September to November 2021. Ship track data consist of a large number of AIS data points containing multiple time series of data such as the ship’s position, speed and heading (Table 1). These data are continuously updated over time to form a dynamic trajectory. However, raw AIS trajectory data often have problems such as noise, missing values, outliers, and data inconsistency, which may affect the training effect and prediction ability of subsequent models. To ensure the quality of AIS data, we adopted a feasible data preprocessing workflow, which has been successfully applied to AIS ship trajectory analysis tasks [25,26]. The workflow includes data standardization and conversion, data cleansing, data filtering, trajectory segmentation, and interpolation and resampling.

1. Data Standardization and Conversion: We normalize continuous numerical features, including longitude, latitude, and ship speed, to eliminate the influence of scale differences on the model training process. We convert UNIX timestamps in the original AIS data into a standard date–time format and sort trajectory points in chronological order to provide a unified temporal reference for subsequent time-series analysis and resampling.
2. Data Cleansing: During the data cleansing process, we must validate the validity of coordinate data and remove invalid values, illegal characters, missing values, or duplicate entries to ensure the independence and consistency of the data. For example, heading values should be restricted to the range of 0 to 360 degrees, and speed values should be non-negative. Heading or speed data outside these ranges should be considered outliers and either corrected or removed.
3. Data Filtering: Based on the experimental requirements of this study, we extract key features, including timestamps, longitude, latitude, speed, and heading, from the AIS data. We remove vessel types and status information that are irrelevant to the research. Furthermore, the data are filtered according to the defined research area, and trajectory points outside the study region are excluded.
4. Trajectory Segmentation: To mitigate the impact of ship berthing, abnormal maneuvers, and AIS signal interruptions on trajectory continuity analysis, we calculate the time interval between consecutive AIS data points of the same ship. When the interval exceeds 6 min, we regard the trajectory as interrupted and divide it into distinct sub-trajectory segments.
5. Interpolation and Resampling: AIS data usually contain missing values and irregular time sampling intervals. To address this issue, we perform interpolation for each sub-trajectory segment separately. We use cubic spline interpolation to reconstruct each sub-trajectory and obtain a continuous trajectory representation. Based on the interpolated results, we resample the trajectory at a fixed interval of two minutes to ensure temporal consistency in the trajectory time series. The dynamic features, such as angular velocity, acceleration, and angular acceleration, are computed by differencing the resampled discrete trajectory sequence.

### 3.2. CNN–SOFTS-Based Framework

The CNN–SOFTS-based framework couples spatial and temporal features for ship trajectory prediction. It employs a CNN module to extract macroscopic spatial patterns from ship trajectories and a SOFTS module to model long-term temporal dependencies in ship motion sequences. Within the SOFTS module, the STAD mechanism aggregates information across all channels to construct a global core representation and redistributes it to individual channels, enabling effective channel interaction with enhanced adaptability and robustness. The spatial features extracted by the CNN are concatenated with the temporal features learned by SOFTS along the feature dimension to form a joint spatiotemporal representation, which is then fed into a fusion regression module to predict future ship trajectories.

#### 3.2.1. CNN Model

**Figure 3:**
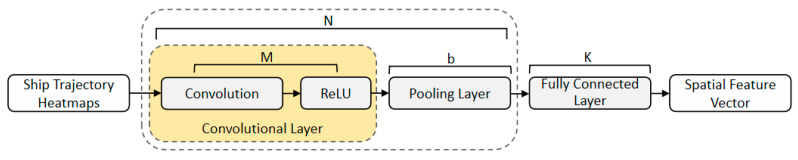
Spatial feature extraction of ship trajectory based on CNN. The daily ship trajectory heatmap is input into a convolutional neural network. Through convolutional layers and pooling operations, the global spatial structure pattern formed by ship navigation is learned, and finally, a 6-dimensional spatial feature vector is output.

In the CNN–SOFTS-based framework, the CNN model is responsible for extracting spatial features. As shown in Figure 3, the core component of CNN is the convolutional block. Each convolutional block contains M convolutional layers and b pooling layers. A typical CNN architecture is formed by stacking N consecutive convolutional blocks and connecting K fully connected layers. Based on input data dimensions, CNN can be divided into one-dimensional CNN and multi-dimensional CNN. One-dimensional CNN is mainly used for text processing. Two-dimensional CNN and three-dimensional CNN are suitable for image and video analysis tasks, respectively. Therefore, this study adopts two-dimensional CNN to process ship trajectory data. This is achieved by mapping trajectory points to longitude–latitude spatial heatmaps to extract spatial distribution features.

Daily ship trajectory heatmaps are input into a CNN model to extract spatial features. The heatmap is constructed at the daily scale to represent the macroscopic spatial distribution of navigation patterns. For each prediction sample, the prediction start time *t*_0_ is used as a truncation point. The spatial heatmap is generated only from historical trajectory points that satisfy *t* < *t*_0_, with no trajectory information after *t*_0_ included. The spatial heatmap is first normalized and resized to a 128 × 128 single-channel grayscale image, and then processed by the CNN spatial encoder, which performs layer-by-layer convolution and pooling operations to learn the global spatial structure patterns formed during ship navigation. The encoder consists of three convolutional blocks, each including a 3 × 3 convolution layer with stride 1 and padding 1, followed by a ReLU activation and a 2 × 2 max-pooling layer with stride 2, with channel dimensions increasing progressively from 1 to 16, 32, and 64. A global average pooling layer is then applied, followed by a fully connected layer that maps the 64-dimensional feature to a 6-dimensional spatial embedding. The model ultimately outputs a 6-dimensional spatial feature vector that captures the dominant macroscopic spatial distribution patterns of daily ship trajectories while maintaining low computational complexity, and this vector serves as the spatial embedding to characterize the overall distribution features of ships within the waterway. In this study, we selected N = 3, M = 1, b = 3, and K = 1.

**Figure 4:**
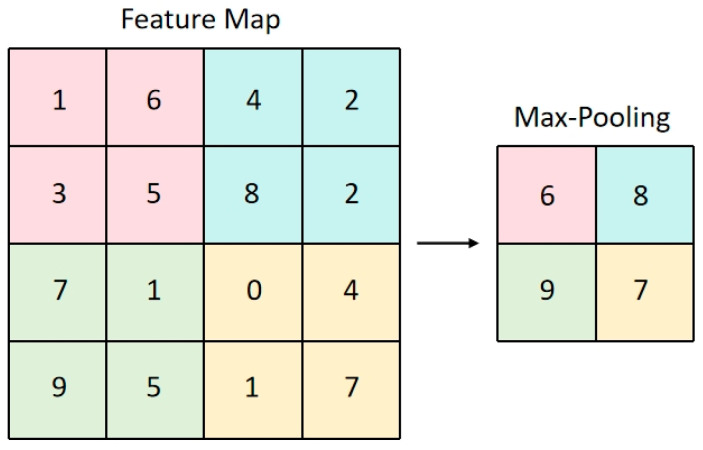
Schematic diagram of the max pooling operation with a 2 × 2 window.

1. Convolutional Layer: As the core component of CNNs, the convolutional layer extracts local features by applying learnable convolution kernels to input data. This operation slides the convolution kernel across the input data, computes the dot product with local regions, and generates feature maps. The convolutional layer commonly employs the Rectified Linear Unit (ReLU) activation function to introduce nonlinear properties. The mathematical calculation of two-dimensional convolution can be expressed by the following equation:
  (1) $$Y(i,j)=\sum_{m=0}^{K_{h}-1}\sum_{n=0}^{K_{w}-1}X(i+m,j+n)\cdot K(m,n)+b$$
  where K denotes the convolution kernel and X represents the input features. Both must be structured as tensors.
2. Pooling Layer: The pooling layer reduces the spatial dimensions of feature maps, decreasing computational complexity while enhancing robustness to variations. Common operations include Max Pooling and Average Pooling. This process traverses feature maps with a fixed-size window, selecting either the maximum or average value within the window region as output. This study employs max pooling to more effectively capture the key regions in the trajectory heatmap. Figure 4 shows an illustration of the max pooling operation using a 2 × 2 window for the original feature data.
3. Fully Connected Layer: The fully connected layer serves as the final stage in CNNs, classifying or regressing features extracted by convolutional and pooling layers. Each neuron in this layer connects to all neurons from the preceding layer, resulting in numerous parameters.

#### 3.2.2. SOFTS Model

##### SOFTS Block

**Figure 5:**
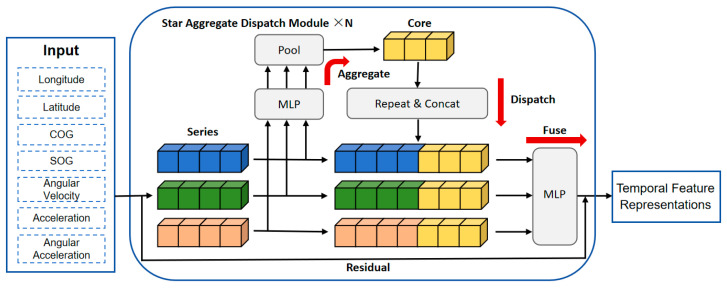
Ship trajectory time feature extraction based on the SOFTS model. The SOFTS model uses its STAD module to model ship motion time series and extract high-dimensional temporal feature representations.

In the CNN–SOFTS-based framework, the SOFTS model is responsible for extracting temporal features. SOFTS is an efficient multivariate time series forecasting model based on MLP, with its architecture illustrated in Figure 5. The model incorporates an innovative STAD module to effectively capture complex correlations among multivariate channels. The STAD module employs a centralized architecture, aggregating all time series into a global core representation, which is then dispatched and integrated into the representations of individual time series. This architecture facilitates information exchange between channels. Compared to traditional models based on distributed architectures, the STAD module offers a significant advantage in computational complexity, allowing SOFTS to maintain high efficiency when processing large-scale datasets while effectively reducing the consumption of computational resources.

We feed the ship motion state sequence, including longitude, latitude, COG, SOG, angular velocity, acceleration, and angular acceleration, into the SOFTS model for temporal modeling. The SOFTS model encodes the input sequence through its STAD modules and outputs a high-dimensional temporal feature representation, which characterizes the temporal dependencies and evolution patterns of ship motion states. In this study, we selected N = 3.

The SOFTS model performs multivariate time series forecasting through the following key techniques:

1. Reversible Instance Normalization: Normalization is a widely used technique aimed at adjusting the scale of data to achieve a uniform range or distribution. This model utilizes a reversible instance normalization method, which is reversible in nature. It centers the sequence to have a mean of zero, scales it to unit variance, and then restores the sequence to its original scale through a denormalization process during the prediction phase. However, normalization may result in the loss of certain data details or information, which could affect the model’s performance. Therefore, the decision to apply normalization should be based on the model’s performance in the specific task at hand.
2. Series Embedding: Series embedding is a special case of patch embedding, where the entire time series is treated as a “patch”. Unlike patch embedding, series embedding does not involve additional segmentation or the introduction of extra dimensions, which results in lower complexity. The model performs series embedding on the retrospective window, mapping each element of the input sequence to $S_{0}={\mathbb{R}}^{C\times d}$, where C represents the number of channels and d is the hidden dimension:
  (2) $$S_{0}=\mathrm{Embedding}(X)$$
3. STAD module: The STAD module employs a star-shaped architecture to enable information exchange between different channels. The sequence embedding is progressively refined through the processing of multiple layers of the STAD module.

(3) $$S_{i}=\mathrm{STAD}(S_{i-1}),\;i=1,2,\ldots ,N$$

##### STAD Block

The STAD module aims to capture dependencies between channels. Existing methods, such as those based on attention mechanisms, typically capture the interactions between channels by calculating the correlations between them. However, as the number of channels increases, the computational complexity of these methods often grows quadratically. When abnormal channels are present, these distributed-structure-based methods may lack robustness, as the abnormal channels can disrupt the correlations between channels. Additionally, when handling non-stationary time series, the correlations between channels are often unstable.

**Figure 6:**
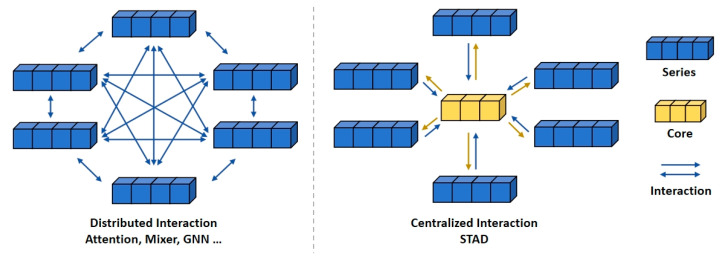
The comparison of the STAD module and several common modules. Compared with conventional distributed-structure modules, the STAD module enhances the model’s robustness in the presence of anomalies and reduces computational complexity.

To address these issues, the STAD module utilizes a centralized architecture to optimize the computation process. It aggregates the information from all sequences to derive a core representation, which captures the global features of all channels. The core information is then dispatched and integrated into each channel, enabling indirect interaction between channels. This method replaces the direct interaction approach based on distributed structures, which is commonly used in traditional modules. Compared to distributed architectures, the interaction pattern of the STAD module significantly enhances the model’s robustness in the presence of anomalies and reduces computational complexity. Figure 6 clearly illustrates the core concept of the STAD module and contrasts it with conventional modules.

Next, the working principles of the STAD module will be explained in detail.

1. The input to the STAD module comes from the sequence representations of each channel. After receiving the sequence representation $S_{i-1}\in {\mathbb{R}}^{C\times d}$ from the previous layer, where C is the number of channels and d is the hidden dimension, the i-th layer of the STAD module processes the input through an MLP and pooling operations to obtain the core representation:
  (4) $$O_{i}=\mathrm{SP}({\mathrm{MLP}}_{1}(S_{i-1}))$$

MLP_1_ is the ${\mathbb{R}}^{d}\mapsto {\mathbb{R}}^{d^{\prime }}$ projection layer, which maps the sequence’s hidden dimension d to the new sequence dimension $d^{\prime }$. This layer consists of two fully connected layers, each with a hidden dimension of d, and uses the GELU activation function. SP refers to stochastic pooling, which combines the advantages of both average pooling and max pooling, and is used to obtain the core representation $O\in {\mathbb{R}}^{d^{\prime }}$.

2. The core representation is integrated into the representation of each sequence:
  (5) $$F_{i}=\mathrm{Repeat}\_\mathrm{Concat}(S_{i-1},O_{i})$$
  (6) $$S_{i}={\mathrm{MLP}}_{2}(F_{i})+S_{i-1}$$

The core representation O is concatenated with each sequence representation using the Repeat_Concat operation, resulting in $F_{i}\in {\mathbb{R}}^{C\times (d+d^{\prime })}$. Then, MLP_2_ (${\mathbb{R}}^{d+d^{\prime }}\mapsto {\mathbb{R}}^{d}$) is used to integrate the concatenated representations and map them back to the hidden dimension d, yielding $S_{i}\in {\mathbb{R}}^{C\times d}$. Additionally, the model introduces a residual connection from input to output.

### 3.3. Evaluation Metrics

In this study, we used three key metrics to evaluate the accuracy of ship trajectory prediction: Mean Squared Error (MSE), Mean Absolute Error (MAE), and the Coefficient of Determination (R^2^). MSE and MAE reflect the model’s prediction accuracy. Smaller values of MSE and MAE indicate higher prediction accuracy. R^2^ is used to assess the model’s fit to the variation in the data, with values closer to 1 indicating better model fit.

We predict four target variables, including longitude, latitude, COG, and SOG, at five output time steps. At each output step, we compute MSE, MAE, and R^2^ for the four variables and then average these metrics with equal weights to obtain the aggregated error for that step. We then average the aggregated errors across the five output time steps and report the resulting MSE, MAE, and R^2^ as the final results. The following are the calculation formulas for the three evaluation metrics:

(7) $$MSE=\frac{1}{n}\sum_{i=1}^{n}{\left(y_{i}-{\hat{y}}_{i}\right)}^{2}$$

(8) $$MAE=\frac{1}{n}\sum_{i=1}^{n}|y_{i}-{\hat{y}}_{i}|$$

(9) $$R^{2}=1-\frac{\sum_{i=1}^{n}{\left(y_{i}-{\hat{y}}_{i}\right)}^{2}}{\sum_{i=1}^{n}{\left(y_{i}-\bar{y}\right)}^{2}}$$

where $y_{i}$ is the true value, ${\hat{y}}_{i}$ is the predicted value, ${\bar{y}}_{i}$ is the mean of the true values, and n is the total number of samples.

The accuracy comparison based on the above evaluation metrics includes two parts:

1. In the comparison of evaluation metrics for ship trajectory prediction over epochs, we compare the proposed method with several advanced time series forecasting methods, including the MLP-based TiDE model [27], the Transformer model, and other deep learning-based models, such as LightTS [28] and FiLM [29].
2. We perform an ablation study on dynamic time variables to analyze their impact on prediction performance.

## 4. Results and Analysis

### 4.1. Experimental Setup

Our experimental setup mainly consists of three components within the CNN–SOFTS-based framework: CNN model setup, SOFTS model setup, and spatiotemporal feature coupling and trajectory prediction setup. (i) The CNN model extracts 6-dimensional macroscopic spatial features from the daily trajectories of ships. (ii) The SOFTS model takes the ship’s longitude, latitude, COG, SOG, angular velocity, acceleration, and angular acceleration as input features. It learns dynamic evolution patterns in the time series to capture the temporal characteristics of ship motion. (iii) An adaptive weighted fusion mechanism integrates temporal and spatial features, with initial weights of 5 for temporal features and 2 for spatial features. This mechanism dynamically optimizes feature weighting during training. The prediction targets are ship longitude, latitude, COG, and SOG.

To ensure the fairness of the experimental results, all models in this study adopt a unified parameter setting. Each model predicts ship positions and motion states over the next five time steps based on the preceding 30 time steps of historical data. Since the trajectories are resampled at 2-min intervals, the five prediction steps correspond to a 10-min prediction horizon. Each model uses the same set of input features to eliminate performance differences caused by feature inconsistency. The data split is performed based on ship identifiers, MMSI. All ships are divided into the training, validation, and test sets at a ratio of 70%:20%:10%, where 70% of the ships are used for model training, 20% for validation, and the remaining 10% for final ship trajectory prediction. Based on the convergence behavior of the training and validation sets, the model reached stable performance at the 20th epoch. Further training did not bring noticeable improvement. Therefore, we set the maximum number of training epochs to 20 to avoid unnecessary computational cost. The loss function used for the models is MSE, which measures the model’s prediction error by calculating the square of the difference between the predicted and true values. The optimizer is the well-performing Adam optimizer, with an initial learning rate set to 3 × 10^-4^. This learning rate is adjusted by a cosine learning rate scheduler to aid the model’s convergence and effectively mitigate issues such as gradient explosion or vanishing gradients during training. The experiments were conducted on a computer using Python 3.11.5, with a 64-bit operating system, 16.0 GB RAM, and an Intel(R) Core(TM) i5-10505 CPU @ 3.20 GHz.

### 4.2. Comparison of Evaluation Metrics for Ship Trajectory Prediction over Epochs

**Figure 7:**
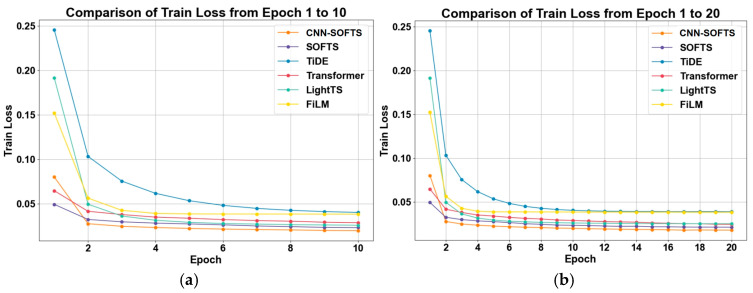
Training loss curves of different models on the training set as a function of epochs. (**a**) Epoch 1–10; (**b**) Epoch 1–20. The CNN–SOFTS-based framework demonstrates faster convergence speed and ultimately achieves the lowest and most stable training loss level.

We compare the training loss curves of different models on the training set, and the results are shown in Figure 7. It can be noted that the training losses of all models decrease rapidly during the initial epochs and subsequently stabilize. This trend shows that the model training has reached preliminary convergence, indicating a stable and effective optimization process. In the early training stage, the CNN–SOFTS-based framework exhibits slightly higher training loss than the SOFTS model. This difference primarily arises because the CNN–SOFTS-based framework requires additional training epochs to learn spatial features extracted by the CNN. As the number of iterations increases, the CNN–SOFTS method demonstrates faster convergence speed and ultimately achieves the lowest and most stable training loss level. These results demonstrate that the CNN–SOFTS-based framework effectively extracts and integrates spatial feature information, thereby significantly enhancing the overall feature representation capability.

**Figure 8:**
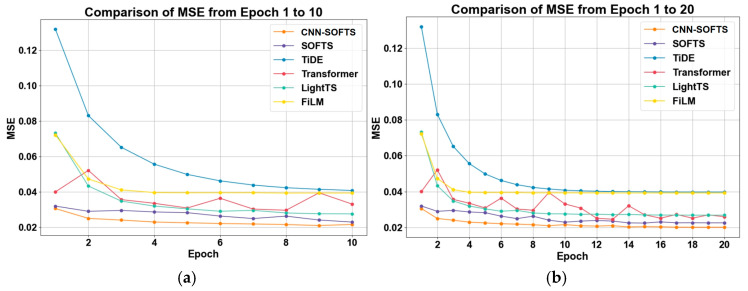
MSE of different models on the validation set as a function of epochs. (**a**) Epoch 1–10; (**b**) Epoch 1–20. The CNN–SOFTS–based framework maintains the lowest MSE values throughout the training process, achieving rapid convergence, especially during the initial training phase.

**Figure 9:**
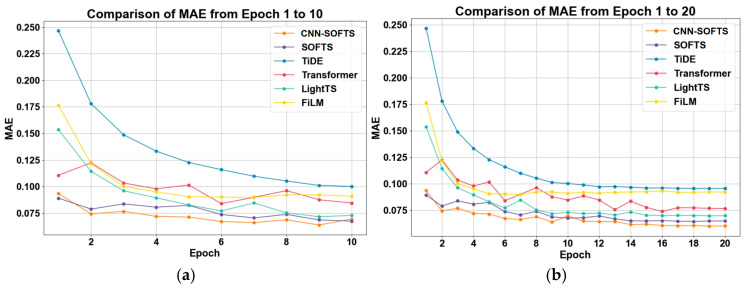
MAE of different models on the validation set as a function of epochs. (**a**) Epoch 1–10; (**b**) Epoch 1–20. The CNN–SOFTS–based framework achieves relatively lower MAE values during the entire training process and reaches the lowest MAE in later stages.

**Figure 10:**
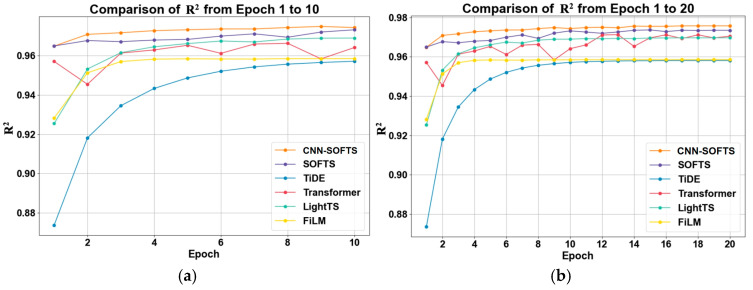
R^2^ of different models on the validation set as a function of epochs. (**a**) Epoch 1–10; (**b**) Epoch 1–20. The CNN–SOFTS–based framework achieves the best performance in R^2^ metrics.

We further evaluated the performance of the CNN–SOFTS–based framework on the validation dataset, and the results are shown in Figure 8. The CNN–SOFTS-based framework demonstrates significant advantages in terms of MSE variation across epochs 1 to 20. The CNN–SOFTS-based framework maintains the lowest MSE values throughout the training process, achieving rapid convergence and stability, especially during the initial training phase. Figure 9 reveals that the CNN–SOFTS-based framework achieves relatively lower MAE values during the entire training process and reaches the lowest MAE in later stages, indicating superior prediction accuracy and stability. As shown in Figure 10, different models display notable differences in R^2^ values during training. Most models experience rapid R^2^ growth within the first five epochs before converging. The CNN–SOFTS-based framework achieves the best performance in R^2^ metrics, starting with higher initial values, maintaining near-constant R^2^ as training progresses, and ultimately reaching the highest value.

The three evaluation curves of the Transformer model all exhibit certain fluctuations (Figure 8, Figure 9 and Figure 10). These fluctuations indicate that the Transformer model has not fully learned the patterns in the data during the early training stages, making it difficult to rapidly adapt to data variations. In contrast, the SOFTS model demonstrates outstanding performance across all metrics. It exhibits superior adaptability and lower error, particularly under shorter training cycles. The advantage of the SOFTS model mainly arises from its unique centralized interaction mechanism, namely the STAD module. This module efficiently captures long-sequence dependencies while significantly reducing computational complexity and achieving faster convergence and higher prediction accuracy. Building on the architectural strengths of SOFTS, the CNN–SOFTS-based framework further integrates the macro spatial features extracted by CNNs. This integration enhances the model’s overall representational capacity, leading to stronger stability and robustness in the later training stages. The experimental results demonstrate that the CNN–SOFTS-based framework outperforms the comparative models in MSE, MAE, and R^2^ metrics. It exhibits superior performance and reliability for inland river ship trajectory prediction (Table 2).

**Figure 11:**
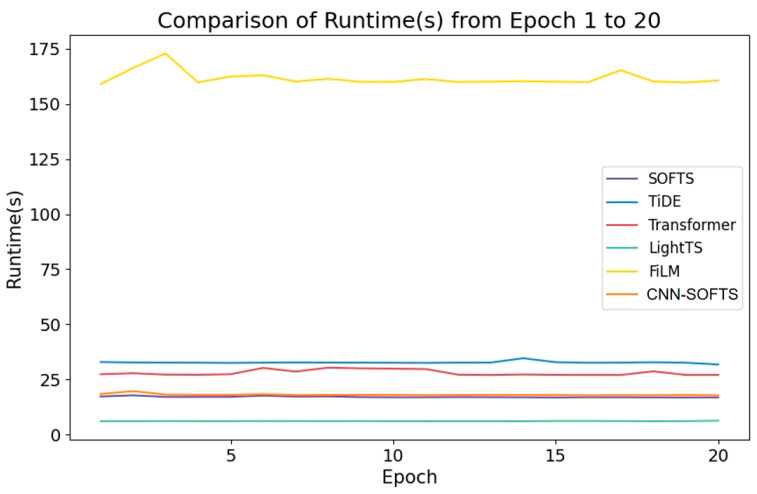
Comparison of runtime of different models over epochs. The CNN–SOFTS–based framework requires approximately 18 s per training epoch, which is only about 1 s longer than the SOFTS model.

Figure 11 presents the training times of different models. With the exception of the FiLM model, the runtime per epoch of all other models is within 50 s. The CNN–SOFTS-based framework requires only approximately 1 s more per training epoch compared to the SOFTS model. Despite the integration of CNN-extracted spatial trajectory features, the CNN–SOFTS-based framework maintains good overall operational efficiency, with a per-epoch runtime of approximately 18 s and minimal fluctuations. Although the LightTS model holds a slight advantage in pure computational speed, the CNN–SOFTS-based framework maintains efficient computation while simultaneously achieving superior prediction performance. This demonstrates the superior overall performance of the CNN–SOFTS-based framework approach.

### 4.3. Comparison of Ship Trajectory Predictions

**Figure 12:**
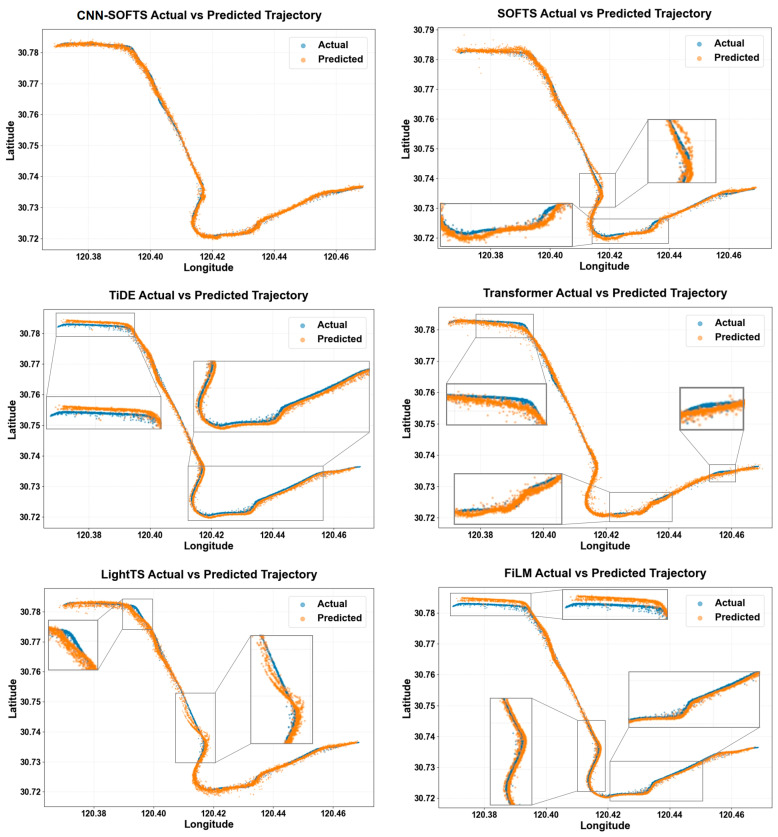
Comparison of actual and predicted trajectory scatter plots for different models. Compared with other models, the CNN–SOFTS-based framework achieves the best performance in trajectory prediction, and its predicted trajectory closely aligns with the actual trajectory.

Figure 12 demonstrates the performance of six methods in the trajectory prediction task, including the proposed CNN–SOFTS-based framework and five models (SOFTS, TiDE, Transformer, LightTS, FiLM). We evaluated the prediction accuracy of each method by comparing the point distributions of predicted trajectories against actual trajectories within the longitude–latitude coordinate system. Overall, the CNN–SOFTS-based framework achieves the best performance in the trajectory prediction task. Its predicted trajectory exhibits close alignment with the actual trajectory, showing minimal deviation between predicted and actual points. Furthermore, the trajectory predicted by the CNN–SOFTS-based framework demonstrates superior overall continuity and smoothness. In contrast, the other models show poorer performance in trajectory prediction. The SOFTS method exhibits significant deviation at trajectory turning segments, which results from its inadequate consideration of macro-spatial features. The introduction of CNN complements the limitations of SOFTS in modeling spatial structures and improves the prediction stability of the model in trajectory turning regions. Methods including TiDE, Transformer, LightTS, and FiLM also demonstrate prediction biases in localized regions, revealing their limitations in complex navigation scenarios.

Overall, the CNN–SOFTS-based framework demonstrates significant advantages in trajectory prediction accuracy and robustness. This is mainly attributed to its comprehensive consideration of spatial and temporal features. By coupling spatial features extracted by the CNN model with temporal features learned by the SOFTS model, the CNN–SOFTS-based framework comprehensively captures ship navigation patterns and behavioral dynamics. This integration enhances the precision and stability of trajectory prediction in curved waterways.

### 4.4. Ablation Study on Dynamic Time Variables

We also investigated the influence of dynamic time variables (angular velocity, acceleration, and angular acceleration) on the prediction performance of the CNN–SOFTS-based framework. The experiments employed two distinct input feature sets for the SOFTS model: one set comprising longitude, latitude, COG, SOG, angular velocity, acceleration, and angular acceleration, and another set utilizing only the four fundamental features: longitude, latitude, COG, and SOG. The effect of dynamic time variables on trajectory prediction is assessed by comparing the two sets of predictions.

**Figure 13:**
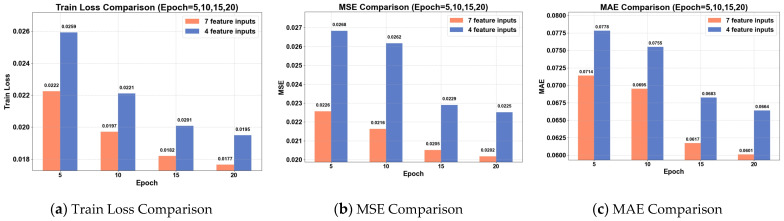
Comparison of training loss and evaluation metrics on the validation set under different feature inputs. At 5, 10, 15, and 20 epochs, the input combination containing seven features consistently outperforms the combination with only four basic features in terms of training loss, MSE, and MAE.

Figure 13 presents the performance of the CNN–SOFTS-based framework under different feature input conditions in terms of training loss and evaluation metrics. The experimental results demonstrate that at 5, 10, 15, and 20 training cycles, the input combination containing seven features consistently outperforms the combination with only four basic features in training loss, MSE, and MAE. As the number of training epochs increases, both feature combinations exhibit decreasing trends in training loss, MSE, and MAE, indicating steady improvement in model prediction accuracy. By the 20th training cycle, the seven-feature combination achieves an approximately 10.22% reduction in MSE and a 9.49% reduction in MAE compared to the four-feature combination. This demonstrates that introducing dynamic time variables enables the model to learn data distributions and evolution patterns more effectively, thereby enhancing its trajectory prediction performance.

## 5. Discussion

This paper presents a CNN–SOFTS-based framework for ship trajectory prediction, which significantly improves the prediction accuracy. Comparative experiments have verified the effectiveness and advantages of the CNN–SOFTS-based framework. However, an analysis of the experimental results reveals some issues and areas for improvement, providing directions for future research.

1. Although the CNN–SOFTS-based framework demonstrates high consistency with actual ship trajectories at most positions, it still exhibits noticeable deviations in specific segments. The larger errors in the predicted trajectory points may be caused by the following factors: (a) In complex waters, such as ports and narrow channels, ships frequently adjust their speed and course. These variable maneuvers increase the difficulty of prediction and may lead to deviations in the results [30]. (b) Adverse weather conditions, such as typhoons and high waves, can cause abnormal deviations in ship behavior. When the model lacks training samples for these specific environments, its prediction accuracy may decrease [31]. (c) The CNN–SOFTS-based framework still has room for improvement in capturing features with abrupt changes. Future research could consider optimizing SOFTS architectures, improving feature extraction methods, and leveraging the strengths of multiple models to further enhance its performance in inland ship trajectory prediction.
2. Current ship trajectory prediction primarily depends on AIS data and does not integrate other sensor information, such as electronic charts, meteorological data, or ocean current data. The lack of multimodal data integration may limit the accuracy of the prediction results [32,33]. While AIS data can reflect the dynamic state of the ship, they cannot comprehensively describe other key factors that may influence ship behavior during navigation. Electronic charts can provide detailed geographical information of the marine environment, meteorological data can reflect the effects of wind, air pressure, and other factors on navigation, and ocean current data can reveal changes in water flow. These factors can significantly impact the ship’s trajectory. Future research will combine real-time meteorological information, ocean current data, and ship status data, among other sources, through multimodal data fusion. This approach will enhance the model’s accuracy and adaptability to diverse navigation conditions, providing more reliable support for maritime safety and marine management.

## 6. Conclusions

We propose a CNN–SOFTS-based framework for ship trajectory prediction. The framework aims to improve prediction accuracy in complex navigation scenarios, such as curved waterways. We first employ a CNN-based spatial encoder to hierarchically abstract spatial density distributions through convolution and pooling operations, thereby learning global spatial structure patterns of ship movements. This encoder emphasizes overall spatial morphology rather than precise individual trajectory points. Second, we employ the SOFTS model to incorporate angular velocity, acceleration, and angular acceleration as input features to characterize ship motion states, which can capture the temporal dependencies of ship motion states from multivariate time series. Finally, the spatial embedding features extracted by the CNN are concatenated with the temporal feature representations learned by SOFTS along the feature dimension to form a joint spatiotemporal representation. The experimental results indicate that the framework excels in prediction accuracy and computational efficiency. It demonstrates promising application potential in practical shipping management and intelligent navigation systems. It can provide reliable trajectory prediction support for route planning, risk warning, and decision support modules, thereby enhancing waterway traffic efficiency and navigation safety.

This study has yielded preliminary results, but there is still room for improvement. Future research will further analyze the trajectory points with large prediction errors, explore the underlying causes, and implement targeted improvements to enhance the model’s prediction accuracy and robustness. In addition, future work may integrate multi-source data, such as meteorological, ocean current, and ship behavior data, to improve the model’s adaptability under varying maritime conditions. These issues will be explored in greater depth in subsequent research to better meet the growing demands in the field of intelligent maritime systems. Future work may explore ship navigation strategies based on predicted trajectories and further compare neural network techniques with fuzzy logic systems [34,35].

## Figures and Tables

**Table 1:** AIS data variables used for model training.

Field Name	Explanation	Example
MMSI	Unique identifier of a ship	413,966,402
Timestamp	AIS data recording time	24 November 2021 8:59:00
Longitude	Longitude of the ship’s current position	120.40180
Latitude	Latitude of the ship’s current position	30.76845
Course Over Ground (COG)	Direction of the ship’s actual movement over ground	168
Speed Over Ground (SOG)	Speed of the ship relative to the ground	2.5

**Table 2:** Evaluation metrics for different time series forecasting models.

Model	Epochs	MSE (× 10^−2^)	MAE (× 10^−2^)	R^2^
CNN–SOFTS	5	2.2563	7.1390	0.9732
SOFTS	5	2.8279	8.2354	0.9683
TiDE	5	4.9873	12.2709	0.9487
Transformer	5	3.0914	10.1526	0.9653
LightTS	5	3.0424	8.2806	0.9662
FiLM	5	3.9539	9.0477	0.9584
CNN–SOFTS	10	2.1626	6.9511	0.9743
SOFTS	10	2.2962	6.7497	0.9732
TiDE	10	4.0824	10.0184	0.9572
Transformer	10	3.3140	8.4578	0.9641
LightTS	10	2.7607	7.2875	0.9690
FiLM	10	3.9371	9.0959	0.9585
CNN–SOFTS	15	2.0513	6.1735	0.9755
SOFTS	15	2.2475	6.4806	0.9737
TiDE	15	3.9903	9.5861	0.9580
Transformer	15	2.7113	7.7656	0.9696
LightTS	15	2.7020	7.0189	0.9696
FiLM	15	3.9273	9.2342	0.9586
CNN–SOFTS	20	2.0181	6.0126	0.9757
SOFTS	20	2.2612	6.4802	0.9735
TiDE	20	3.9810	9.5511	0.9581
Transformer	20	2.6015	7.6487	0.9705
LightTS	20	2.6877	6.9756	0.9697
FiLM	20	3.9268	9.2078	0.9586

## Data Availability

Restrictions apply to the availability of these data. The datasets presented in this article are not readily available because the datasets are subject to administrative restrictions.
